# Convergent Evolution of Boats with Sails

**DOI:** 10.1038/s41598-020-58940-5

**Published:** 2020-02-17

**Authors:** A. Bejan, L. Ferber, S. Lorente

**Affiliations:** 1 Duke University, Department of Mechanical Engineering and Materials Science, Box 90300, Durham, NC 27708-0300 USA; 2 Lockheed Martin Space, 12257 South Wadsworth Boulevard, Littleton, CO 80125 USA; 3 Villanova University, Department of Mechanical Engineering, Villanova, PA 19085 USA

**Keywords:** Evolution, Anthropology, Evolutionary theory, Energy science and technology, Renewable energy, Engineering, Mechanical engineering

## Abstract

This article unveils the geometric characteristics of boats with sails of many sizes, covering the range 10^2^–10^5^ kg. Data from one hundred boat models are collected and tabulated. The data show distinct trends of convergent evolution across the entire range of sizes, namely: (i) the proportionality between beam and draft, (ii) the proportionality between overall boat length and beam, and (iii) the proportionality between mast height and overall boat length. The review shows that the geometric aspect ratios (i)–(iii) are predictable from the physics of evolution toward architectures that offer greater flow access through the medium.

## Introduction

Nature impresses us with images, changes and tendencies that repeat themselves innumerable times even though “similar observations” are not identical to each other. In science, we recognize each ubiquitous tendency as a distinct *phenomenon*. Over the centuries, our predecessors have summarized each distinct phenomenon with its own law of physics, which then serves as a ‘first principle’ in the edifice of science. A principle is a ‘first principle’ when it cannot be deduced from other first principles.

This aspect of organization in science is illustrated by the evolution of thermodynamics to its current state^1,2^. For example, 150 years ago the transformation of potential energy into kinetic energy and the conservation of “caloric” were fused into one statement—the first law of thermodynamics—which now serves as a first-principle in physics. It was the same with another distinct tendency in nature: everything flows (by itself) from high to low. This, the phenomenon of one-way flow, or irreversibility, was summarized in another statement at the same time—the second law of thermodynamics—which serves as another first-principle in physics.

Why do the most common occurrences need such a long time to be recognized as natural tendencies (phenomena), and even longer to be recorded in physics with a short statement, a first principle? Because the evolution of the human mind is an integral part of the evolution of the human, to adapt and survive while struck by *unexpected* dangers, environmental, animal, and human. The first thing that we question is the unusual (the “surprise”, which means being grabbed from above, as if in the claws of a predator). Questioned the least are the most common observations, the familiar, the not threatening. This is why *new* questions in science are rare.

Nowhere is the human approach to science more evident than in the face of the natural phenomenon of evolutionary organization^1,3^. Images, morphing images, impress us constantly, yet the most common images go unnoticed. For example, the oneness of natural tree-shaped architectures of the inanimate realm (e.g., river basins) and the animate realm (e.g., human lungs, city traffic) is evident and intriguing. Recent articles are drawing attention to phenomena of evolution that are general and belong in physics^3–23^. This literature shows that such phenomena are predictable. Examples are the architectures of lungs^24^ and corals^25^, the life span and life travel of animals, vehicles, rivers and the winds^26^, the round cross sections of all jets and plumes^27^, the dendritic architecture and S-shaped history of dendritic solidification such as snowflakes^28^, the arrow of time of evolutionary organization^29^, and the fact that humans prefer unwittingly certain shapes and proportions, from the shapes of the Egyptian pyramids^30^ and the shapes of fires (piles of fuel)^31^, to the golden-ratio shape of drawings, images and text.

Because of its physics basis, the phenomenon of evolution can be imagined the way it happened, in retrospect. Further ahead along this line, evolution can be not only predicted but also witnessed in our life time, for example, by observing technological evolution. The geometric similarity of modern commercial aircraft^32^, like the similarity of helicopters^33^ and automobiles^34^, shows that human movement on the world map is facilitated by the generation and persistence of certain shapes and structures.

**Figure 1:**
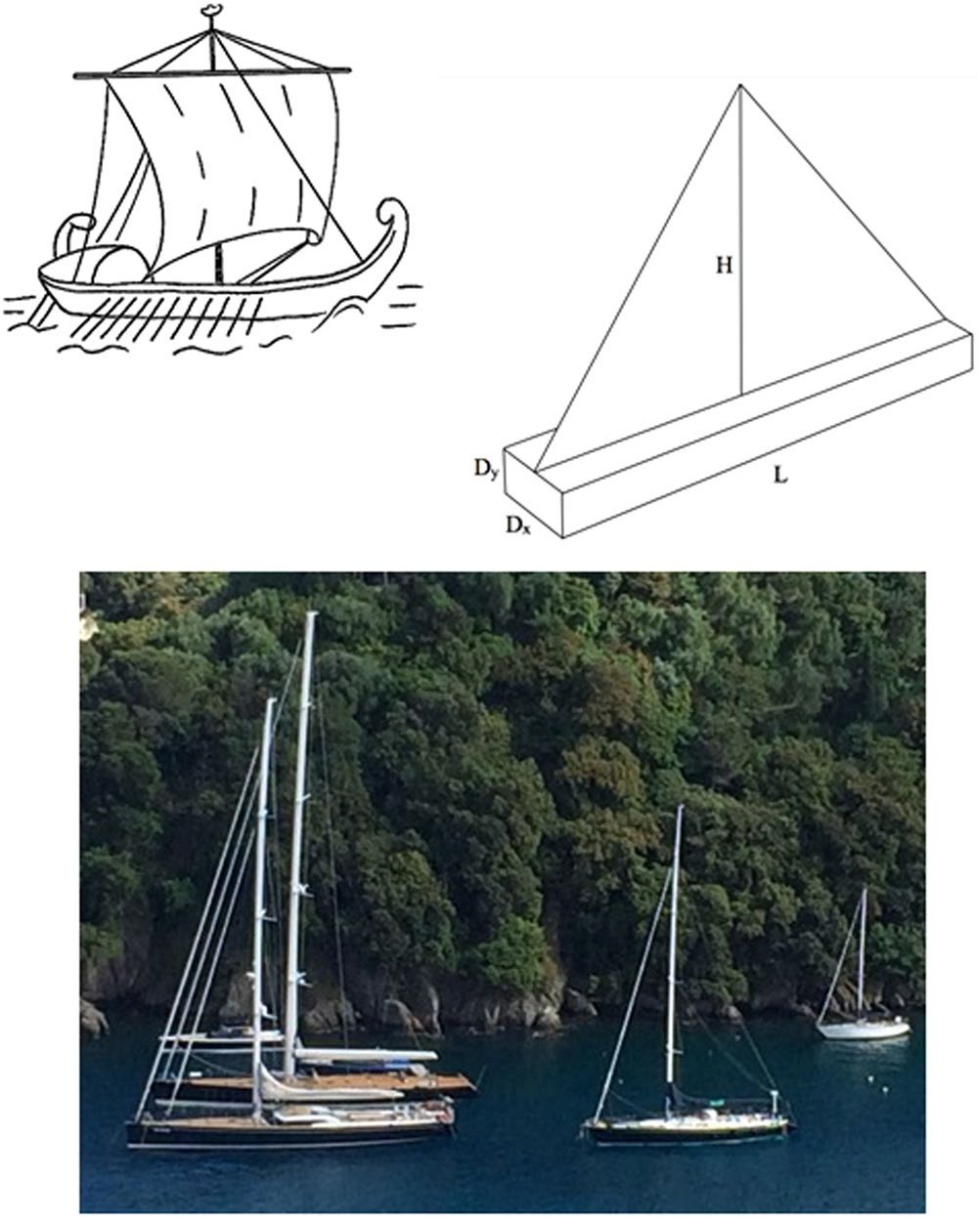
The geometric similarity of boats with sails, clockwise: ancient Egyptian galley, the essential length scales of the moving body, and modern sailboats (photo: Adrian Bejan).

In this paper we strengthen this message by questioning an evolutionary phenomenon of technology that is evident (Fig. 1) but goes unquestioned. Why do boats with sails look the same? They have sails that are roughly as tall as the length of the hull. They have hulls that are longer than they are wide. Furthermore, they are submerged to a depth that is greater when the hull is wider. The boat has been this way since antiquity. Even more intriguing is that the large boat looks just like the small boat. Why?

## Theory

The reason for all these observations is the human tendency to move more easily on earth^1,3^. The vehicle architecture that emerges is a reflection of the urge of all its builders and users to move more easily, to have greater access to the surroundings. In recorded times, this tendency gave birth to artifacts (vehicles) in which people encapsulate themselves to acquire greater access. From this idea, the convergent evolution of all boats with sails is deducible.

**Figure 2:**
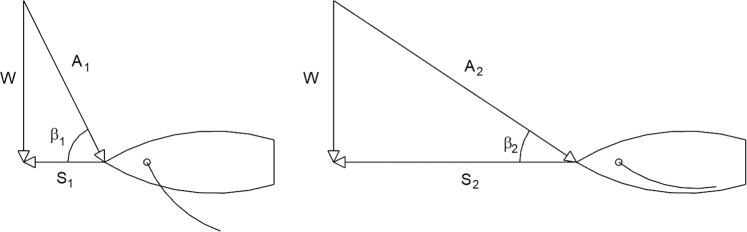
Two cases showing the relationship between wind speed (W), apparent wind speed (A), boat speed (S) and apparent wind angle (β). The sails are trimmed to account for apparent wind angle.

The boat moves horizontally with the speed V_w_ on the surface of the water. The wind with the speed V_a_ engages the sail with the force,

1 $${{\rm{F}}}_{{\rm{a}}} \sim {{\rm{C}}}_{{\rm{D}}}({\rm{HL}}/2)\frac{1}{2}{\rho }_{{\rm{a}}}{{\rm{V}}}_{{\rm{a}}}^{2}$$

where C_D_ ~ 1 is the drag coefficient, ρ_a_ is the air density, and (HL/2) is the sail area: H is the height of the mast, and L is the hull length. The driving force F_a_ is matched by the drag force experienced by the hull against the water,

2 $${{\rm{F}}}_{{\rm{w}}} \sim [{{\rm{C}}}_{{\rm{D}}}{{\rm{D}}}_{{\rm{x}}}{{\rm{D}}}_{{\rm{y}}}+{{\rm{C}}}_{{\rm{f}}}({{\rm{D}}}_{{\rm{x}}}+2{{\rm{D}}}_{{\rm{y}}}){\rm{L}}]\frac{1}{2}{\rho }_{{\rm{w}}}{{\rm{V}}}_{{\rm{w}}}^{2}$$

where D_x_ is the hull width, D_y_ is the depth of the submerged portion of the hull, ρ_w_ is the water density, and C_f_ is the skin friction coefficient for turbulent flow, the order of magnitude of which is C_f_ ~ 10^−2^ ^35^. Note the two terms in the square brackets: the first accounts for the drag experienced by the hull frontally, as a blunt body, and the second is due to the fluid friction along the hull. As we show later in the discussion of Fig. 2, the forces that propel the boat can vary depending on the angle of attack.

From the balance between F_w_ and F_a_ emerges the ratio V_w_/V_a_, which is larger when the quantity in square brackets in Eq. (2) is smaller. This quantity varies in accord with the two aspect ratios of the configuration, D_x_/D_y_ and D_x_/L, subject to the displaced volume of water (D_x_D_y_L), which is fixed because it is dictated by the total weight of the boat. It is easy to show analytically that the quantity in the square brackets in Eq. (2) is minimum when

3 $$\frac{{{\rm{D}}}_{{\rm{x}}}}{{{\rm{D}}}_{{\rm{y}}}} \sim 2$$

and

4 $$\frac{{\rm{L}}}{{{\rm{D}}}_{{\rm{x}}}} \sim \frac{{{\rm{C}}}_{{\rm{D}}}}{2{{\rm{C}}}_{{\rm{f}}}} > 1$$

This settles the question of the shape of the hull and that of most fish: they should be slender in profile, and relatively round when viewed in cross section.

What about the shape of the sail? When the aspect ratios of Eqs. (3, 4) apply, the drag force in the water [Eq. (2)] becomes

5 $${{\rm{F}}}_{{\rm{w}}} \sim 3{(\frac{2{{\rm{C}}}_{{\rm{f}}}}{{{\rm{C}}}_{{\rm{D}}}})}^{2}{\rm{L}}\,{\rho }_{{\rm{w}}}{{\rm{V}}}_{{\rm{w}}}^{2}$$

From the balance between Eqs. (1) and (5) we deduce that

6 $$\frac{{\rm{H}}}{{\rm{L}}} \sim 3{(\frac{2{{\rm{C}}}_{{\rm{f}}}}{{{\rm{C}}}_{{\rm{D}}}})}^{2}\frac{{\rho }_{{\rm{w}}}}{{\rho }_{{\rm{a}}}}{(\frac{{{\rm{V}}}_{{\rm{w}}}}{{{\rm{V}}}_{{\rm{a}}}})}^{2}$$

which in view of C_D_ ~ 1, C_f_ ~ 10^−2^, and ρ_w_/ρ_a_ ~ 10^3^, becomes

7 $$\frac{{{\rm{V}}}_{{\rm{w}}}}{{{\rm{V}}}_{{\rm{a}}}} \sim {(\frac{{\rm{H}}}{{\rm{L}}})}^{1/2}$$

In the evolutionary pursuit of higher boat speeds V_w_, the height of the sail approaches the length of the hull. Expressed in terms of scale analysis, the conclusion is that H and L must have the same scale because V_w_ and V_a_ represent the same scale (no wind, no travel; fast wind, fast travel).

With the three aspect ratios now predicted (D_x_/D_y_, L/D_x_, H/L) the evolutionary direction of the boat model selected in Fig. 1 is complete, and can be drawn in three dimensions. The shape viewed from above is L/D_x_, while from the front and from the side it is respectively D_x_/D_y_ and H/L. There are only three shapes because the configuration of the simplest model (Fig. 1) has only three degrees of freedom. Each of these shapes refers to the external look of the model.

**Figure 3:**
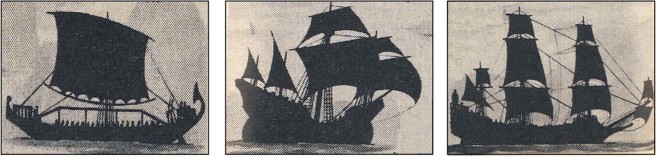
The evolution of boats with sails over the past two thousand years: Egyptian galley, Columbus and Napoleon.

Boat designs are more complicated because in addition to external shape they also have internal structure. The internal structure has additional geometric details, which have increased in number during boat evolution. Three frames from this evolutionary sequence are aligned chronologically in Fig. 3, from two thousand years ago (Egyptian galley) to Columbus crossing the Atlantic (1492) and Napoleon’s navy (1800). In antiquity the internal structure was the simplest: one mast supporting one sail. Over time, the sails and the masts became more numerous as the speed and carrying capacity of the boats increased for the benefit of the people who constructed, owned and operated them. Modern monohulled sailboats align more with the Egyptian galley in that there is typically a single mast. The fastest monohulls in the world have a single mast and achieve maximum speeds with 2–3 sails.

In Fig. 3 the three boats are presented in frames of the same size in order to stress two additional points. In time, the complexity of the architecture increases as the internal structure morphs. Yet, in every frame the external shapes are the same as those that we predicted for the simplest model without internal structure (Fig. 1).

Features of internal structure can be predicted by continuing the analysis started in Eqs. (1–7). Assume that the lone sail in Fig. 1 is supported by one mast, which is modeled as an elastic rod of height H and diameter d. The mast bends under the horizontal force received from the sail, which is F_a_, Eq. (1). The mast is a beam in pure bending, because it is slender enough so that its slenderness H/d exceeds 50.

The highest stresses occur at the base of the mast, where the bending moment is maximum and of order F_a_H. This moment is balanced by the moment due to the nonuniform distribution of stresses in the mast cross section. The stresses are tensile on the forward (convex) side of the mast in bending, and compressive on the aft (concave) side. If the material is such that σ is the order of magnitude of the highest allowable (tensile and compressive) stresses at the base, then the bending couple in the cross section is of order σd^2^ × d, where the σd^2^ are the forces of the couple (tensile and compressive, both aligned with the mast) and d is the arm of the couple, which is transversal to the mast. From the requirement of rotational equilibrium, F_a_H ~ σd^3^, we obtain

8 $$\frac{{{\rm{d}}}^{3}}{{{\rm{H}}}^{2}{\rm{L}}} \sim \frac{{\rho }_{{\rm{a}}}{{\rm{V}}}_{{\rm{a}}}^{2}}{\sigma }$$

which, in view of Eq. (7), becomes

9 $$\frac{{\rm{d}}}{{\rm{H}}} \sim {(\frac{{\rho }_{{\rm{a}}}{{\rm{V}}}_{{\rm{a}}}^{2}}{\sigma })}^{1/3}{(\frac{{{\rm{V}}}_{{\rm{a}}}}{{{\rm{V}}}_{{\rm{w}}}})}^{2/3}$$

To summarize, the predicted evolutionary design has the three external shapes discussed previously, plus one internal shape, d/H, the mast slenderness. One formula, Eq. (9), governs the evolution of this technology, past and future. A stronger material (larger σ) makes a more slender (thinner, lighter) mast, which in turn decreases the dead weight of the vessel (and the submerged portion of the hull), reduces hull friction and increases the boat speed.

## Comparison with Current Designs

In the modern era, physics principles have played a guiding role in the improvements that have been made in the design of boats with sails. The icon of the central role of physics in boat design is Euler’s entry^36^ in the 1727 contest for the King’s prize for the solution to the nautical problem to determine the best way to place the masts on vessels, and the relation between their positions and the number and height of the masts. Since then, fluid dynamics and naval engineering grew as scientific domains, as did naval vessel technology before and after the advent of steam power^37–42^. In this section we compare the current state of sail boat architecture with the design features predicted theoretically.

**Figure 4:**
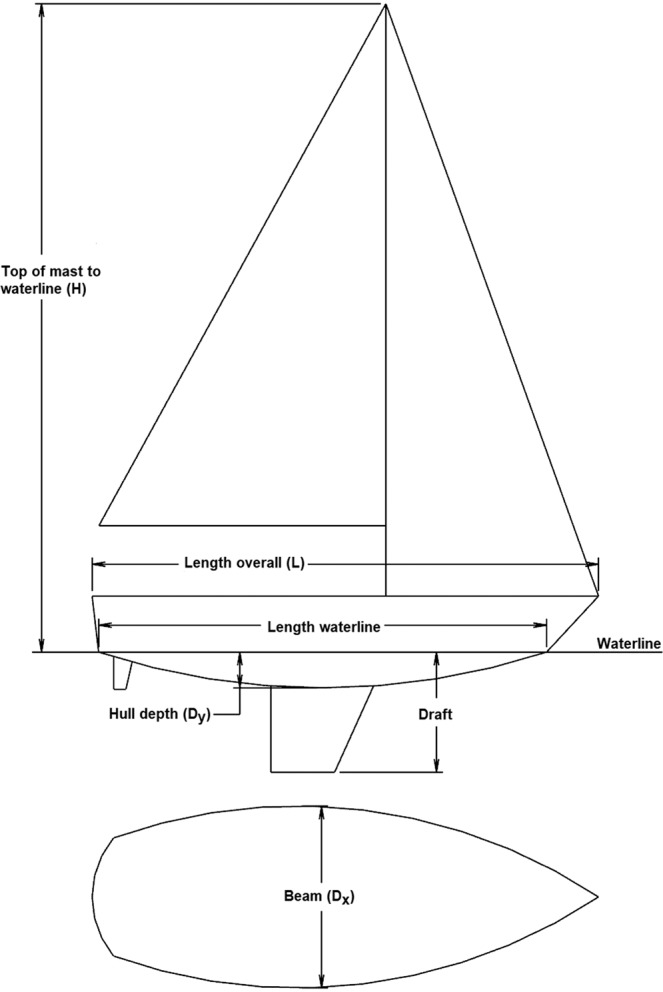
The main dimensions used in yacht construction.

Table 1 consists of 96 single hulled sailboats with a variety of models, years, dimensions, weights, and designs^43^. The variables D_x,_ D_y,_ L, and H correlate to Beam, Hull Depth, Overall Length, and Height, respectively, which are defined in Fig. 4. The displacement [kg] is the mass of the boat, which is equal to the mass of the water that is displaced by the boat.

**Table 1:** Sailboat model data.

Model	Year	Length Overall (L)	Length Waterline (L)	Beam (Dx)	Sail Area (HL/2)	Draft (max)	Displacement	Ballast	PHRF Rating	Hull Depth (Dy)	Height (H)	Hull Shape	Rig Setup
		(m)	(m)	(m)	(m2)	(m)	kg	kg	s/nm	(m)			
Aerodyne 47	2001	14.2	12.83	4.37	87.14	1.83	11508	4686	42	0.6	18.43	Fin w/bulb & spade rudder	Fractional Sloop
Alajuela 33	1977	10.06	8.38	3.25	53.42	1.45	6124	2132	198	0.82	13.4	Fin w/rudder on skeg	Cutter
Alberg 35	1961	10.59	7.32	2.95	50.63	1.57	5715	2404	201	0.57	13.04	Long keel	Masthead Sloop
Alerion	1996	6.1	5.21	2.08	20.44	1.07	862	354	249	0.25	9.74	Fin w/bulb & spade rudder	Fractional Sloop
Archabault 31	2009	9.55	8.3	3.23	50.91	1.9	3150	1350	111	0.47	13.94	Fin w/spade rudder	Fractional Sloop
B-25	1989	7.62	6.48	2.54	25.36	1.52	907	363	141	0.29	11.05	Lifting keel	Fractional Sloop
Baba 40	1980	12.14	10.52	3.91	80.27	1.83	13154	5445	183	0.63	16.85	Long keel	Cutter
Balboa 26	1969	7.8	6.35	2.44	27.5	1.52	1633	544	225	0.37	10.32	Swing keel	Masthead Sloop
Baltic 37	1978	11.28	8.31	3.66	59.36	2.03	6169	2776	114	0.57	15.48	Fin w/spade rudder	Masthead Sloop
Baltic 51	1984	16.76	14.44	4.92	136.1	2.19	17500	7321	51	1.08	22.3	Fin w/spade rudder	Masthead Sloop
Beneteau 49	2005	15.09	13.31	4.5	95.13	1.75	12935	4300	54	0.74	18.38	Fin w/bulb & spade rudder	Fractional Sloop
Bianca 111	1975	11.13	9.4	3.2	68.28	1.98	6087	2794	93	0.62	16.07	Fin w/spade rudder	Fractional Sloop
Black Watch 37	1965	11.28	7.62	3.2	55.46	1.55	7031	1905	183	0.7	14.95	Fin keel	Masthead Yawl
Boothbay Harbor OD	1938	6.4	6.02	1.68	21.09	1.07	953	408	252	0.29	8.49	Fin keel	Fractional Sloop
Bravura Sportster 29	2001	9.12	7.87	2.9	46.08	2.03	1814	816	72	0.32	14.5	Fin keel	Fractional Sloop
Brewer 12.8	1983	12.8	10.29	4.11	82.4	2.74	10818	4990	126	0.77	16.93	Keel/CB	Cutter
Bridges Point 24	1985	7.32	5.69	2.36	35.12	1.04	1789	1270	243	0.67	9.84	Long keel	Fractional Sloop
Bristol 47.7	1979	14.33	11.35	4.01	89.93	3.35	15722	6804	114	0.77	18.27	Keel/Cbrd.	Masthead
Buccaneer 220	1978	6.86	5.87	2.41	19.04	0.91	1111	431	234	0.29	9.37	Fin w/trans. hung rudder	Fractional Sloop
Bullseye	1914	4.79	3.82	1.78	13.01	1.65	612	340	360	0.56	6.9	Long keel w/trans. hung rudder	Fractional Sloop
Buzzard’s Bay 15	1899	7.47	4.57	2.06	30.75	1.68	1103	454	219	0.37	8.2	Keel/Cbrd.	Gaffhead
C&C 115	2005	11.51	10.06	3.63	72.55	2.03	5352	1905	66	0.52	16.9	Fin w/bulb & spade rudder	Fractional Sloop
Cal 24 (Hunt)	1983	7.52	6.1	1.3	24.25	1.3	1497	533	219	0.32	9.56	Fin w/spade rudder	Masthead Sloop
Cambria 44	1985	14	11.07	4.11	88.53	1.8	12973	5216	87	0.7	19.2	Fin w/rudder on skeg	Cutter
Cape Dory 25	1973	7.57	5.49	2.21	24.34	0.91	1814	771	261	0.5	9.33	Long keel	Masthead Sloop
Carrera 290	1992	8.89	8.21	2.88	38.93	1.7	1338	603	99	0.14	14.28	Fin w/bulb & spade rudder	Fractional Sloop
Cartwright 40	1975	12.19	9.35	3.43	73.3	1.78	10660	4763	153	1.19	15.31	Long keel	Cutter
D&M 22	1971	6.71	5.72	2.57	23.6	1.68	1259	454	258	0.36	9.71	Fin w/trans. hung rudder	Masthead Sloop
Dehler 39	1996	11.89	10.69	3.82	95.87	1.95	7001	2945	81	0.45	17.16	Fin w/bulb & spade rudder	Fractional Sloop
DK 46	2002	14.1	12.35	4.1	134.89	3	8650	4300	−30	0.54	22.95	Fin w/bulb & spade rudder	Fractional Sloop
Dolphin 24 (S&S)	1959	7.36	5.79	2.34	27.59	1.58	1928	748	246	0.46	10.12	Keel/Cbrd.	Masthead Sloop
Dufour 2800	1977	8.25	6.75	2.93	30.19	1.46	2751	900	204	0.39	10.9	Fin w/spade rudder	Masthead Sloop
Electra (Person)	1960	6.86	5.11	2.13	21.18	0.91	1361	589	288	0.39	8.96	Fin keel	Masthead Sloop
Endeavour 32	1976	9.75	7.7	2.97	43.11	1.28	5307	2268	190	0.58	13.21	Fin w/rudder on skeg	Masthead Sloop
Ericson 31-2	1967	9.22	7.11	2.9	38.09	1.47	3538	1361	168	0.46	13.29	Fin w/spade rudder	Masthead Sloop
Esprit 37	1977	11.28	9.63	3.51	61.96	1.77	7711	3039	150	0.7	17.37	Fin w/rudder on skeg	Cutter
Etchelles ODR	1966	9.3	6.71	2.13	27.96	1.37	1508	987	120	0.4	11.71	Fin w/rudder on skeg	Fractional Sloop
Express 37	1984	11.3	9.4	3.51	59.36	2.21	4445	2087	72	0.43	15.43	Fin w/spade rudder	Masthead Sloop
Farr 36 OD	2002	11	10	3.57	51.28	2.59	3059	1596	0	0.28	17.81	Lifting keel	Fractional Sloop
Fast Passage 39	1976	12.04	10.21	3.61	74.23	1.68	9526	3402	132	0.77	16.82	Fin w/rudder on skeg	Cutter
Figaro Solo	1989	9.14	8.4	3.25	48.77	1.8	2400	900	48	0.34	13.22	Fin w/bulb & spade rudder	Fractional Sloop
Finngulf 37	2004	11.25	10	3.5	64.64	2	6500	2250	84	0.48	16.85	Fin keel w/bulb	Fractional Sloop
Flying Tiger 10 m	2005	9.95	9.24	2.79	49.89	2.32	1984	870	54	0.32	14.61	Lifting keel	Fractional Sloop
Gladiator 24	1958	7.32	6.1	2.29	25.73	1.22	1746	930	258	0.48	10.83	Fin keel	Fractional Sloop
Grampian 30	1969	9.07	7.77	2.9	39.48	1.42	3901	1755	192	0.73	13.38	Fin w/spade rudder	Masthead Sloop
Hallburg Rassy 31	1992	9.56	7.74	3.32	46.17	1.73	4572	2032	198	0.57	13.51	Fin w/spade rudder	Fractional Sloop
Hanse 385	2011	11.4	10.4	3.88	67.26	1.99	7600	2200	99	0.53	16.98	Fin w/bulb & spade rudder	Fractional Sloop
Harbor 20	1997	6	5.18	2.13	20.44	1.07	816	408	225	0.22	9.17	Fin w/bulb & spade rudder	Fractional Sloop
Harpoon 6.2	1979	6.2	4.82	2.44	17.56	1.12	771	249	240	0.25	8.33	Fin w/trans. hung rudder	Fractional Sloop
Henderson 30	1997	9.4	8.76	3	44.59	2.13	1746	794	45	0.29	15.83	Lifting keel	Fractional Sloop
Hunter 336	1995	10.21	8.71	3.56	53.23	1.37	5003	1860	147	0.43	17.02	Fin w/bulb & spade rudder	Fractional Sloop
Irwin 23	1968	7.01	5.64	2.44	23.78	1.75	1452	680	252	0.38	9.4	Keel/CB & spade rudder	Masthead Sloop
Islander 29	1968	8.86	7.11	2.69	36.42	1.12	3538	1134	234	0.51	11.35	Fin keel	Masthead Sloop
Islander Packet 320	1998	10.13	8.23	3.58	51.65	1.3	6124	2722	198	0.77	14.84	Long keel	Cutter
IW-31	1968	9.24	6.71	2.69	33.17	1.65	3538	1542	192	0.64	10.84	Fin w/rudder on skeg	Masthead Sloop
J/111	2010	11.1	9.97	3.29	61.22	2.19	4350	1595	42	0.39	15.86	Fin w/bulb & spade rudder	Fractional Sloop
J/160	1996	16.06	14.48	4.42	127.83	2.13	14152	5443	−9	0.7	22.1	Fin w/bulb & spade rudder	Fractional Sloop
Jonmeri 48	1988	14.52	12.2	4.74	109.16	2.51	15640	7100	75	0.81	20.2	Fin w/spade rudder	Masthead Sloop
Kirby 25	1974	7.67	6.32	2.67	26.85	1.27	1429	522	177	0.2	10.68	Fin w/spade rudder	Fractional Sloop
Knutson35	1955	10.67	7.62	3	53.05	1.47	7711	2431	195	0.83	14.7	Fin keel	Fractional Sloop
Lafitte 44	1978	13.51	10.82	3.86	89.83	1.93	12701	5130	126	0.77	17.4	Fin w/rudder on skeg	Cutter
Lager 40	1984	12.09	10.13	3.86	74.41	2.18	5579	3039	60	0.58	17.05	Fin w/spade rudder	Fractional Sloop
Little Harbor 60	1995	18.42	14.55	4.93	139.81	3.63	31979	10433	48	1.2	22.42	Keel/Cbrd.	Cutter
Luders 16	1933	8.03	4.98	1.75	20.81	1.22	1338	726	216	0.45	9.96	Long keel	Masthead Yawl
Medalist 33	1965	10.06	7.34	3.05	42.36	1.6	5307	1905	234	0.87	13.89	Fin w/spade rudder	Masthead Sloop
Mistress 32	1969	9.7	6.9	2.84	41.34	1.85	3400	1500	195	0.63	11.25	Fin w/rudder on skeg	Masthead Sloop
Monhegan 48	1998	14.82	11.43	4.19	96.71	1.52	12746	5443	72	0.76	19.09	Keel/Cbrd.	Masthead Sloop
Moody 45 DS	2010	13.72	12.93	4.57	97.92	1.99	13600	4300	108	0.81	20.52	Fin w/bulb & twin rudders	Fractional Sloop
Mumm 36	1993	10.92	9.68	3.61	59.83	2.23	3697	1588	42	0.38	16.8	Fin w/bulb & spade rudder	Fractional Sloop
Najad 373	1999	11.3	9.75	3.65	59.46	1.9	8300	3100	156	0.64	17.1	Fin w/rudder on skeg	Masthead Sloop
Nevins 40	1955	12.19	8.38	3.43	68.65	2.31	9979	2313	168	0.83	14.33	Keel/Cbrd.	Masthead Yawl
Newport 28	1974	8.46	7.62	2.9	36.7	1.3	3175	1452	195	0.58	12.64	Fin w/spade rudder	Masthead Sloop
Nordic 44	1980	13.36	10.8	3.94	85.38	2.13	10546	4237	84	0.65	18.78	Fin w/rudder on skeg	Masthead Sloop
Ohlson 36	1958	10.97	7.62	2.84	n/a	1.85	6260	2268	189	0.86	12.56	Fin keel	Masthead Sloop
Oyster 485	1994	14.78	11.43	4.27	89.18	2.13	17033	590	84	0.84	18.08	Fin w/rudder on skeg	Cutter
Puma 23	1971	6.87	5.39	2.21	22.76	1.18	1347	675	258	0.43	9.77	Fin w/rudder on skeg	Masthead Sloop
Quickstep 24	1976	7.29	5.79	2.41	24.06	1.04	1814	862	240	0.36	9.39	Fin w/rudder on skeg	Masthead Sloop
Ranger 33	1969	10.11	8	2.92	49.14	1.52	4763	2041	156	0.45	13.39	Fin w/spade rudder	Masthead Sloop
Redwing 30	1967	9.23	6.63	2.68	37.53	1.37	3383	1647	195	0.77	10.86	Fin w/spade rudder	Masthead Sloop
Rhodes 41	1961	12.44	8.53	3.12	67.35	1.75	8528	3663	171	0.87	14.05	Long keel	Masthead Sloop
Sabre 386	2004	11.79	9.91	3.86	69.12	2.08	7688	2903	84	0.6	16.45	Fin w/bulb & spade rudder	Masthead Sloop
Sabre 426	2002	12.95	10.97	4.09	85.47	2.08	10886	3810	69	0.63	17.34	Fin w/bulb & spade rudder	Masthead Sloop
Saga 43	1996	13.18	11.86	3.66	88.44	1.91	9000	3538	81	0.58	18.93	Fin w/bulb & spade rudder	Masthead Sloop
Sailmaster 22	1963	6.71	5.03	2.13	21.27	1.8	1656	635	294	0.33	8.96	Keel/Cbrd.	Masthead Sloop
Seafarer 45	1961	13.74	9.24	3.35	83.42	1.96	11340	4196	156	1.04	15.85	Fin keel	Masthead Sloop
Seascape 27	2011	7.99	7.99	2.54	45.99	1.95	1150	550	105	0.26	12.25	Keel/CB w/twin rudders	Fractional Sloop
Swan 77	1992	24.01	18.38	6	261.14	3.4	51001	18000	−12	1.41	32.11	Fin w/spade rudder	Masthead Sloop
Tartan 3800	1994	11.58	9.45	3.79	61.78	1.62	7258	3175	114	0.64	16.44	Fin w/bulb & spade rudder	Masthead Sloop
Tayana 55	1983	16.76	14	4.9	129.97	1.98	21954	7963	54	0.74	20.77	Fin w/rudder on skeg	Cutter
Thunderbird	1958	7.92	6.17	2.3	28.71	1.46	1656	694	213	0.33	10.97	Fin w/spade rudder	Fractional Sloop
Ultimate 20	1994	6.35	5.49	2.44	22.57	1.52	499	204	144	0.16	9.72	Lifting keel	Fractional Sloop
Viper 830	1996	8.44	7.62	2.59	38.83	2.18	1134	700	66	0.23	12.94	Lifting keel	Fractional Sloop
Whistler 48	1982	14.53	12.27	4.24	108.6	1.78	16288	6196	102	0.93	19.39	Sheel keel	Cutter
X-442	1993	13.51	11.2	4.13	88.81	2.29	9662	4300	36	0.63	18.58	Fin w/bulb & spade rudder	Masthead Sloop
Yankee 26	1974	7.92	6.3	2.64	27.87	1.45	2420	974	264	0.6	12.94	Fin w/rudder on skeg	Masthead Sloop

PHRF is the performance indicator, which stands for Performance Handicap Racing Fleet. PHRF is a handicapping standardization that equates the performance of different boats. It is designed to rate the boat design characteristics only, and is impartial to the talent of the skipper and crew. With this in mind, the skipper and crew who sail the best overall race from boat handling and tactical perspective should be awarded as the victors.

Boats are given PHRF ratings based on empirical data including dimensional characteristics, materials, past race finishes, similar boats scaling, and comparisons to other handicap systems. The PHRF system also accounts for three ranges of wind conditions (light, moderate, heavy breeze) by utilizing distinct constants for the ratings formula. Like all handicapping systems, PHRF is imperfect due to the opportunity to inject subjectivisms but is the most-widely accepted handicapping systems in the U.S.

For the scope of this article, it should be known that the lower or negative ratings correlate to faster boats. For instance, the fastest boat in Table 1 is the DK 46 with a PHRF rating of 30, and the slowest is the Bullseye with a rating of 360. In moderate conditions, the DK 46 should be 1.75 times faster boat-for-boat than the Bullseye when utilizing the correction factor formula. The correction factor is then applied to the overall time, generating a “corrected time” that generates a one-to-one comparison between the different boats. The boat with the lowest corrected time is the winner of the race.

**Figure 5:**
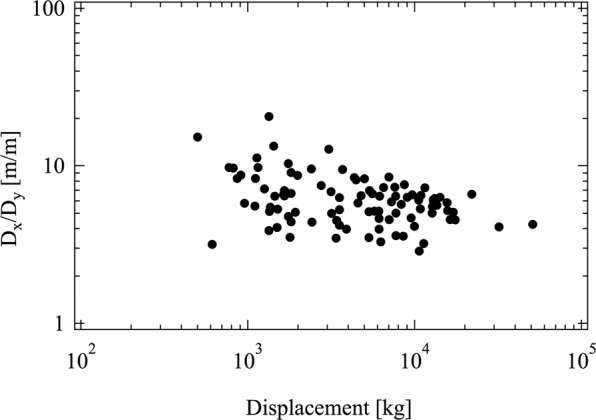
The ratio D_x_/D_y_ (beam/draft) according to the data presented in Table 1.

**Figure 6:**
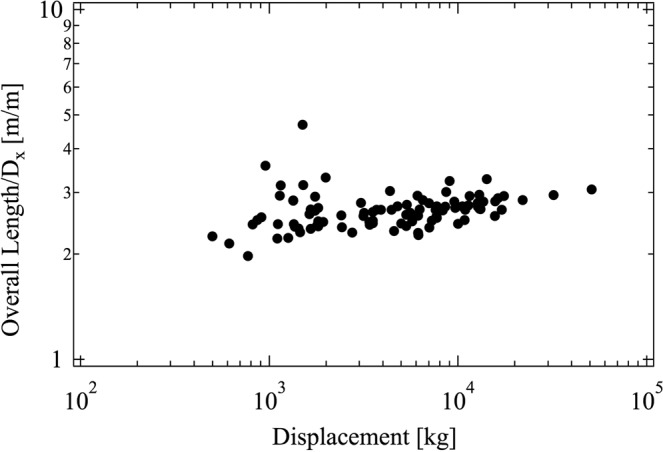
The ratio L/D_x_ (overall length/beam) according to the data presented in Table 1.

**Figure 7:**
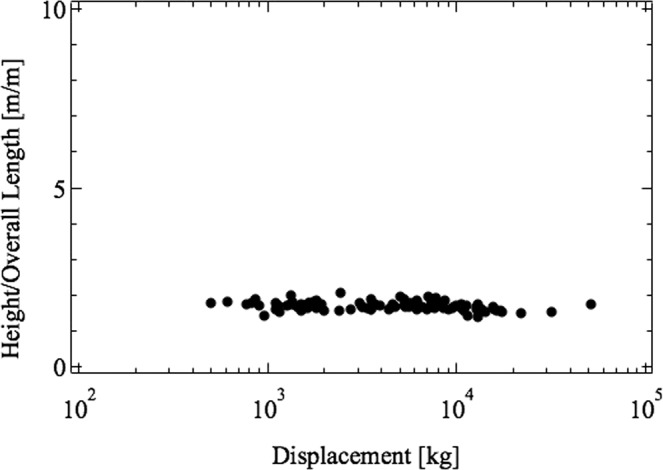
The ratio H/L (height/overall length) according to the data presented in Table 1.

Plotted in Figs. 5–7 are the actual measurements extracted from Table 1. The three figures show the aspect ratios D_x_/D_y_, L/D_x_ and H/L versus the boat displacement. Noteworthy is that the three aspect ratios do not depend on the displacement. This means that the main aspects of the configuration did not change over time, as the displacement increased in history (cf. Fig. 3).

The results provided by Eqs. (3), (4) and (7) predict the convergent evolution observed in Figs. 5–7. The geometrical ratios that define the shape of the boats with sails are permanent characteristics over the ages and cultures.

Due to the number of unique designs, calculating the exact performance of a yacht is dependent on a significant number of intrinsic properties as well as external factors. Different boats achieve peak performances at specified degrees from the wind direction in specific conditions. ‘Degrees’ refers to the boat pointing angle relative to the true wind direction. Ultimately, as the boat moves faster and increases the apparent wind velocity (wind speed plus boat speed), the apparent wind angle becomes small relative to the true wind angle.

The fastest boats in the world are almost always sailing ‘upwind’ (with sails pulled in closer to the hull mid-line) because they can generate significant boat speed relative to the wind speed. This phenomenon is depicted in Fig. 2. Note the two distinct instances, where the boat speed (S_1_ < S_2_) and the angle of attack (β_1_ > β_2_) change, while the true wind speed W is constant. The apparent wind vector A increases with the boat speed when the boat direction and the true wind vector do not change. The sail’s (or airfoil’s) leading edge will point in the direction of the apparent wind to generate lift. As the boat speed continues to increase, the apparent wind angle moves forward until the sail is pulled to centerline (cf., Fig. 2), at which point the boat has reached is maximum speed for its relative boat angle from the true wind direction.

There are various calculations to generate boat speed using a characteristic dimension. One example is the critical velocity or ${{\rm{V}}}_{{\rm{c}}}\cong 1.25\,{({\rm{LWL}})}^{1/2}$. This formula follows from the fact that the bow of a sailboat moving through the water produces a transverse wave, and at critical velocity the wave extends the length of the waterline, which essential traps the boat in the trough, not allowing it to escape the transverse wave, and so capping its velocity. There are various circumstances that can allow a boat to go faster than V_c,_ such as “surfing” external waves or utilizing hydro-foils to lift the hull out of the water.
